# The Effect of Oyster Shell Powder on the High-Temperature-Properties of Slag-Ceramic Powder-Based Geopolymer

**DOI:** 10.3390/ma16103706

**Published:** 2023-05-13

**Authors:** Gui-Yu Zhang, Sihwan Lee, Yi Han, Xiao-Yong Wang

**Affiliations:** 1 Department of Integrated Energy and Infra System, Kangwon National University, Chuncheon-si 24341, Republic of Korea; zhangguiyu@kangwon.ac.kr (G.-Y.Z.); hanyii@kangwon.ac.kr (Y.H.); 2 Graduate School of Environmental Studies, Nagoya University, Furocho, Chikusa Ward, Nagoya 464-8601, Aichi, Japan; shany@nuac.nagoya-u.ac.jp; 3 Department of Architectural Engineering, Kangwon National University, Chuncheon-si 24341, Republic of Korea

**Keywords:** alkaline activator, phase transition, microscopic analysis, size changes

## Abstract

There is a lack of scientific understanding of adding an oyster shell powder (OSP) to geopolymer concrete. The purpose of this study is: (1) to evaluate the high-temperature resistance of the alkali-activated slag ceramic powder (CP) mixture added with OSP at different temperatures, (2) to address the lack of application of environmentally friendly building materials, and (3) to reduce solid waste of OSP pollution and protect the environment. OSP replaces granulated blast furnace slag (GBFS) and CP at 10% and 20% (based on binder), respectively. The mixture was heated to 400.0, 600.0, and 800.0 °C after curing for 180 days. The results of the experiment are summarized as follows: (1) The thermogravimetric (TG) results indicated that the OSP20 samples produced more CASH gels than the control OSP0. (2) As the temperature increased, the compressive strength and ultrasonic pulse velocity (UPV) both decreased. (3) Fourier transform infrared spectroscopy (FTIR) and X-ray diffraction (XRD) results reveal that the mixture undergoes a phase transition at 800.0 °C, and compared with the control OSP0, OSP20 undergoes a different phase transition. (4) The size change and appearance image results indicate that the mixture with added OSP inhibits shrinkage, and calcium carbonate decomposes to produce off-white CaO. To sum up, adding OSP can effectively reduce the damage of high temperatures (800.0 °C) on the properties of alkali-activated binders.

## 1. Introduction

The most common human-made material in use today is cement. Cement serves as a concrete binder and creates stunning structures for human civilization, and simultaneously, its production process emits a large amount of CO_2_ [1,2,3]. Cement production accounts for about 7% of the world’s CO_2_ emissions [4]. In addition, 1700 kg of raw materials are consumed to make 1000 kg of clinker [5]. Currently, the large-scale use of cement is primarily challenged by sustainability, durability, overuse of natural resources, and CO_2_ emissions [6,7]. Recently, environmentally friendly alkali-activated binders have been developed to alleviate problems associated with cement production. Alkali-activated materials (AAM) are green and sustainable alternatives to Portland cement (PC). Industrial waste has been used to partially or completely replace cement with AAMs. The application of alkali activation to industrial waste can significantly reduce the demand for cement and directly reduce CO_2_ emissions. AAMs widely used by researchers include metakaolin, fly ash, and granulated blast furnace slag (GBFS).

With the acceleration of urbanization and renewal of buildings, a large amount of construction waste has been generated worldwide. The main components of construction waste include hardened concrete and ceramics (tiles, tableware, sanitary ware, etc.). Ceramic waste can be finely applied after it is ground into ceramic powder (CP), with a particle size similar to that of cement [5,8,9]. CP was employed as a starting material for alkali activation in prior work [10].

Recently, the mariculture industry has experienced considerable growth worldwide [11,12], particularly in oyster aquaculture. Because of their exquisite flavor, customers are becoming more and more accustomed to eating oysters. In Spain, shellfish generate 80,000 tons of waste each year [12]. Oyster shells are garbage generated by the catering industry, and oyster shells account for approximately 90% of the total mass of oysters [13]. This means that for every kilogram of oyster meat consumed, nine times the waste will be produced. Most of this waste is not utilized and is instead dumped in landfills or dumped into the ocean, having enormous negative effects on the ecosystem [14]. After oysters are thrown, microbial decomposition takes place at specified times and temperatures, producing highly poisonous gases, including NH_3_ and H_2_S [14,15]. This can cause serious public health concerns.

The majority of prior research on oyster shell powder (OSP) as a building material has been on how its usage as an additional cementitious material in place of cement affects the qualities of cement concrete [16,17,18]. These findings indicate that the main component of oyster shells is calcium carbonate and that its chemical properties are very similar to those of natural calcium carbonate minerals. In [16], the feasibility of using OSP and GBFS as cement substitutes to prepare sustainable concrete was investigated. They found that the addition of OSP accelerated the hydration reaction in the first 24 h of the mixture. In [17], a mixture of OSP instead of limestone was studied. They found that OSP was a suitable substitute for limestone. In [18], the feasibility of recycling shellfish waste in concrete was investigated. They found that shellfish waste was not reactive in concrete and served only as a filler material. Liao et al. [19] studied the influence of OSP as a fine aggregate on the fluidity of mortar. They found that increasing the OSP replacement rate decreased the fluidity of the mortar. Song et al. [20] studied the effect of OSP as an aggregate on the durability of concrete. They found that adding OSP to concrete improved its water resistance and corrosion resistance. Liao et al. [21] studied the effect of OSP content on the properties of cement-metakaolin mortar. Considering mechanical properties, durability and drying shrinkage, they believe that 8% OSP is the best dosage. Several studies have investigated the effect of added calcium carbonate minerals on the performance of AAM. The reactivity of calcium carbonate minerals in alkaline solutions has been examined in a previous study [22]. They believed sodium silicate reacts with calcium carbonate minerals, which have a range of crystal shapes, to produce sodium carbonate and CSH. In [23], the properties of alkali-activated binders (fly ash and slag) mixtures containing limestone were investigated. They suggested that limestone could provide the nucleation sites for reaction products, and that the presence of limestone slightly accelerates the reaction process.

A small number of studies have investigated the effect of adding OSP to AAMs [24,25,26]. The effectiveness of a pumice-based geopolymer paste containing calcined oyster shell powder and reclaimed concrete was examined in [24]. They found that recycled concrete and calcined oyster shell powder could provide Ca^2+^ to participate in the reaction to form CASH, which improved the mechanical properties of the mixture. In the literature [25], calcined oyster shells were used as alkaline activators to study the alkali-activated slags. They found that low doses of calcined oyster shell powder helped refine the pores and improve the mechanical properties of AAMs. A further study [26] reported that bauxite and calcined oyster shells could partially replace pozzolanic ash in the synthesis of pozzolan-based polymers. Their study found that a small amount of bauxite or calcined oyster shells, instead of pozzolan, reduced the initial setting time and increased at 28-day compressive strength.

After reviewing the previously reported literature, the main limitations of previous works are summarized as follows. First, some research has been conducted on materials involving added calcium carbonate. However, most research in the past has focused on the effect of OSP on the performance of cementitious concrete. Second, little research has been conducted on AAMs containing OSPs. Third, the effect of OSP on alkali-activated GBFS and CP high-temperature resistance has not been studied to the authors’ knowledge.

To fill this research gap, we evaluated the high-temperature resistance of AAMs with added OSP. Second, oyster shells discarded by the catering industry, ceramic waste produced during building destruction, and blast furnace slag (a by-product of the recycling industry) can produce high-temperature-resistant materials. This is beneficial in terms of its environmental friendliness. In addition, adding OSP to the AAM reduces the use of cement and GBFS, thereby reducing energy usage and CO_2_ emissions.

## 2. Experiment Preparation

### 2.1. Materials

**Figure 1:**
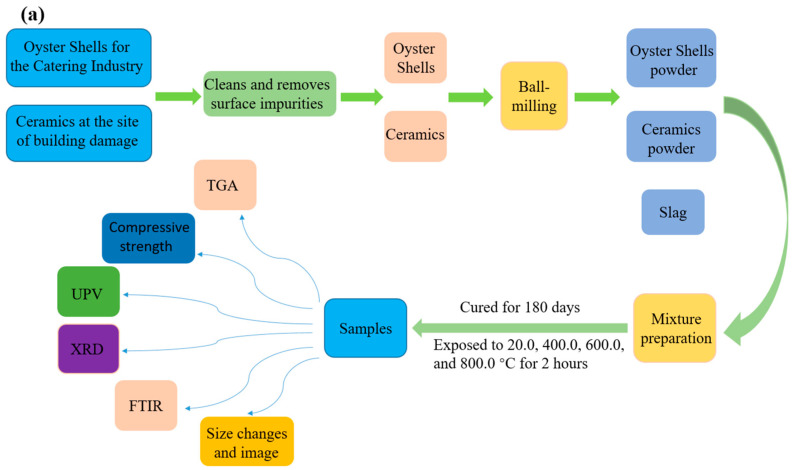

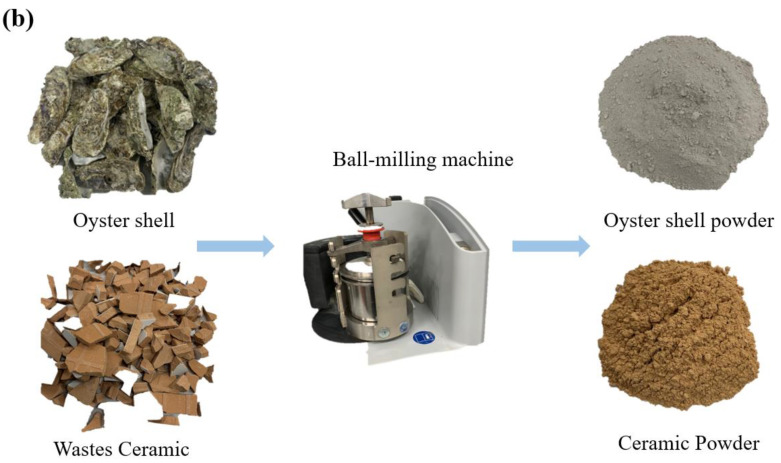
(**a**) Flowchart of the experimental plan.; (**b**) Preparation process of oyster shell and ceramic powders.

Three waste raw materials, GBFS, CP, and OSP, were used in this study for the preparation of triple-base-activated materials. When pig iron is manufactured, GBFS is a waste slag released from a blast furnace. The GBFS used in this study was provided by the Asian Cement Company in Seoul, Korea. We recycle discarded oyster shells from the catering industry. That is because the restaurant industry produces vast amounts of oyster shells each year, putting pressure on the environment [13,14]. Collection of ceramic waste from building destruction. This is because the main waste generated during the destruction of buildings is concrete and ceramics [27]. The ceramic waste and oyster shells were washed first to remove surface impurities. After that, an oven was used to dry the ceramic waste and oyster shells for 24 h at 105 °C. Finally, the samples were crushed and ground with a ball mill to produce CP and OSP. The balling time is 30 min, and the instrument used is Planetary Ball Mill PM 100. Figure 1a is a flowchart of the experimental plan. Figure 1b shows the preparation process of the OSP and CP in the laboratory. Table 1 lists the results of the X-ray fluorescence analysis of their chemical compositions. The main oxide composition of GBFS and OSP included CaO, and the main oxide composition of CP was SiO_2_. According to calculations, the OSP used in this study contained 97.88% calcium carbonate and was comparable to the data reported in the literature [17].

**Figure 2:**
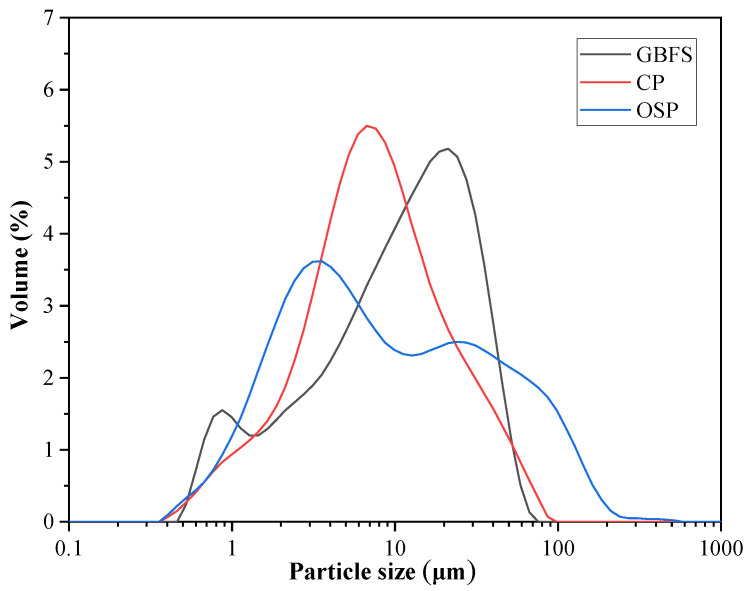
Particle size distributions of GBFS, CP, and OSP.

**Figure 3:**
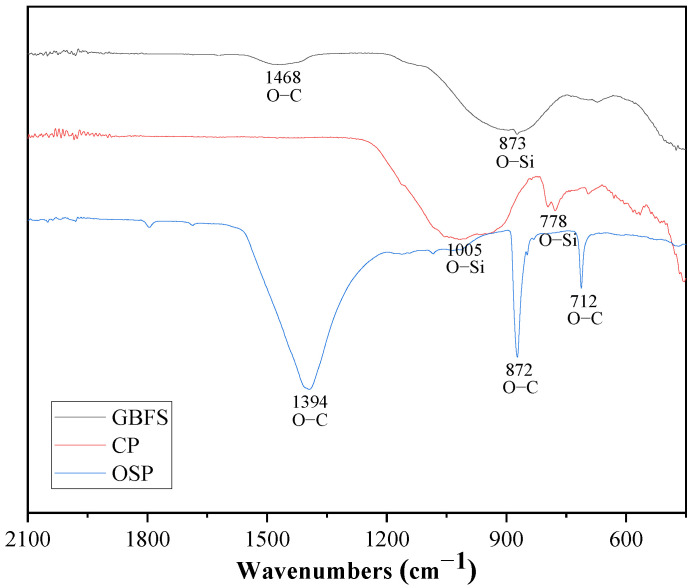
FTIR spectra of GBFS, CP, and OSP.

Figure 2 displays the results of measuring the particle size distribution of GBFS, CP, and OSP using a particle size analyzer (Model Mastersizer 3000, Malvern Instruments Ltd, London, UK). Their median particle sizes were 12.3, 7.93, and 4.55 μm, respectively. The vibrational signatures of chemical bonds in raw materials can be studied using FTIR spectroscopy. The FTIR (PerkinElmer, Waltham, MA, USA) spectra of the GBFS, CP, and OSP are shown in Figure 3. The absorption peak of the O–C bonds in OSP was particularly obvious. This is the same as the result of XRF, the compound in OSP is relatively single. In addition, combined with the literature [16], the single substance here is CaCO_3_. Shells are usually not made of pure calcium carbonate because they would be too brittle. Only a small percentage of proteins (<5%) are then gluing the calcium carbonate lamellae. The FTIR spectra or TGA measurements can find evidence of the presence of proteins. The effect of the presence of proteins on the properties of geopolymer should be investigated in future work. It is worth noting that absorption peaks of O–Si bonds exist in the raw material GBFS, in addition to the few absorption peaks of O-C bonds. This indicates that there is a small amount of CaCO_3_ in the GBFS, which is the reason for the CO_3_^2−^ hydrotalcite phase in the alkali-activated product.

The mixed alkali activator used consisted of liquid sodium silicate solution (Water-glass: SiO_2_ = 27–31%, Na_2_O = 8–11%, and water = 60–63%) and solid sodium hydroxide granules (NaOH: GR grade; purity 98%). NaOH particles were added to a water-glass solution to achieve the desired mixed activator modulus (Ms, SiO_2_/Na_2_O = 1.2) [28]. The solution was stirred using a magnetic stirrer to dissolve the sodium hydroxide particles. Dissolving sodium hydroxide pellets generates a lot of heat; therefore, the beaker must be covered with a thin film during the stirring process to prevent water from evaporating. The resulting mixed alkali solution was left to stand for 6 h to achieve sufficient cooling. Deionized water was added to achieve the desired liquid/binder ratio. The resulting mixed alkali solution was utilized to boost the mixture’s reactivity.

### 2.2. Mixture Ratio

Three sets of mixtures were prepared for this study, all with liquid/binder (L/B) ratios of 0.5 and L/(BFS + CP) ratios of 0.5, 0.556, and 0.625. Table 2 shows the mixing ratios of GBFS, CP, OSP, and alkaline activators for all mixtures. In the mixture, GBFS and CP were replaced with OSP at replacement rates of 10.0 and 20.0%, respectively. In order to determine the mechanical properties of the mixture, the amount and concentration of alkali were determined in advance through preliminary experiments. The mixtures were named OSP0, OSP10, and OSP20. The alkaline activator is 4% of the mass of GBFS, CP, and OSP, as Na_2_O remained unchanged.

### 2.3. Mixture Preparation and Heating Scheme

The materials (GBFS, CP, OSP, and mixed alkali solution) were weighed in proportion. The materials were mixed with a mechanical stirrer and poured into cube molds of dimensions 50 mm and prisms molds of dimensions 40.0 mm × 40.0 mm × 160.0 mm. The mold was opened after 24 h. In order to avoid moisture loss and sample carbonation in the air, the mixture was wrapped in a plastic film and subsequently sealed and cured. Then, the mixture was placed in a curing room at 20.0 ± 2.0 °C for six months.

The mixture is cured for six months. The mixture was then put into a muffle furnace and heated at 6 °C/min from room temperature to 400.0, 600.0, and 800.0 °C. The mixture was naturally cooled in a furnace to room temperature after being heated to the desired temperature for one hour.

### 2.4. Testing Method

#### 2.4.1. Thermogravimetric

TG (SDT Q600, TA Instruments, New Castle, DE, USA) was used to characterize the aged 180-day mix. At a 15 °C/min heating rate, about 25 mg of powder was heated from room temperature to 950 °C. The nitrogen flow used for the experiments was 1.2 L/h. Each sample was tested independently three times to ensure data reproducibility.

#### 2.4.2. Compressive Strength Development

Compressive strength tests were performed on mixtures that were cured for 180 days and exposed to 20, 400.0, 600.0, and 800.0 °C. The sample used was a 50.0 mm cube mixture. The mixtures were tested within 24 h. Select a mixture sample with a relatively flat surface to avoid greater errors. A loading rate of 0.6 kN/s was used until the mixture was broken. Three mixtures exposed to different temperatures were selected for testing, and the average value is the final result.

#### 2.4.3. Ultrasonic Pulse Velocity

UPV (Pundit Lab, Proceq, Schwerzenbach, Switzerland) tests were performed on mixtures cured for 180 d and exposed to 20, 400.0, 600.0, and 800.0 °C. The sample size used for the test was a prism of 40.0 mm × 40.0 mm × 160.0 mm. Test 3 samples and the average value were calculated [29].

#### 2.4.4. Crystal Phase X-ray Diffraction

XRD (Panalytical, Almelo, The Netherlands) analysis was performed on mixtures cured for 180 d and exposed to 20, 400.0, 600.0, and 800.0 °C. The powder mixtures were measured using Kα Cu X-ray radiation at 4.000 V and 0.04 A. We scanned the mixtures 6–8 times from 5° to 60° in 0.02° increments. Calibration was performed with corundum for each scan. 

#### 2.4.5. Fourier Transform Infrared Spectroscopy

FTIR (PerkinElmer, Waltham, MA, USA) analysis was performed on mixtures cured for 180 d and exposed to 20.0, 400.0, 600.0, and 800.0 °C. The samples were anhydrous ground with isopropanol and dried at 45 °C for 10 min. The spectra were gathered in the range of 500–4000 cm^−1^ with a resolution of 0.4 cm^−1^ [30].

#### 2.4.6. Image and Size Changes

The surface of the mixture was observed using a camera (macroscopic image) and microscope (mesoscopic image). Using a Vernier caliper, the apparent size of the mixture cured for 180 days and exposed to 20.0, 400.0, 600.0, and 800.0 °C was measured. Three samples of the same composition at the same temperature for measurement were selected, and their average values were calculated.

## 3. Experimental Results

### 3.1. TGA

**Figure 4:**
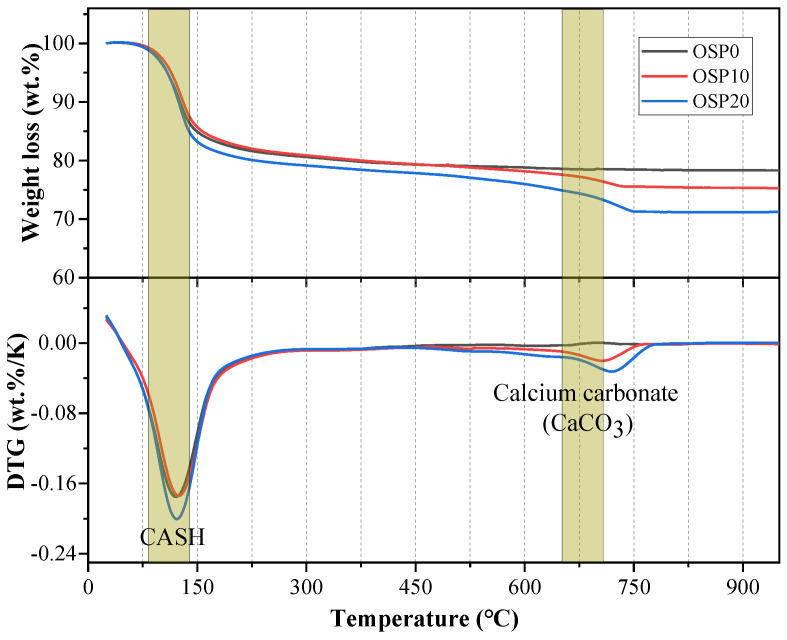
TGA curves of OSP0, OSP10, and OSP20 samples at 180 days.

After 180 d of curing, the OSP0, OSP10, and OSP20 samples’ thermogravimetric and differential thermogravimetric (TG-DTG) curves are displayed in Figure 4. The TG-DTG curves range from room temperature (approximately 22 °C) to 950 °C. After analyzing the TG curves, all samples showed significant mass loss at 90–150 °C due to dehydration of the hydration products [31]. Notably, the mass loss peaks due to calcium carbonate decomposition at around 650 °C for OSP10 and OSP20 samples exhibit a significant enhancement compared to OSP0. This is due to the increase in the amount of OSP added. For the temperature range at which the reaction products of OSP0, OSP10, and OSP20 undergo mass loss upon heating, please refer to the literature [32].

About 120 °C is the primary DTG peak, mainly due to the evaporation and dehydration of water from geopolymers [32,33,34]. After 180 d of curing, the mass loss peak due to dehydration of the geopolymer gel gradually increased as the amount of OSP substitution increased to 20%. This is due to the involvement of calcium carbonate in OSP in the alkali activation reaction and the formation of more CASH gels [22]. As a result, the OSP20 sample produced more CASH gel, a hydration product, than the OSP0 sample. The mass loss is increased by the decomposition of calcium carbonate at temperatures between 500 and 850 °C, which heats up to create calcium oxide and release carbon dioxide. The amount of OSP that is replaced is correlated with weight loss in this temperature range. More specifically, the mass loss of calcium carbonate increases with the increase of OSP content. This is because the main ingredient of OSP is calcium carbonate.

### 3.2. Compressive Strength Development

**Figure 5:**
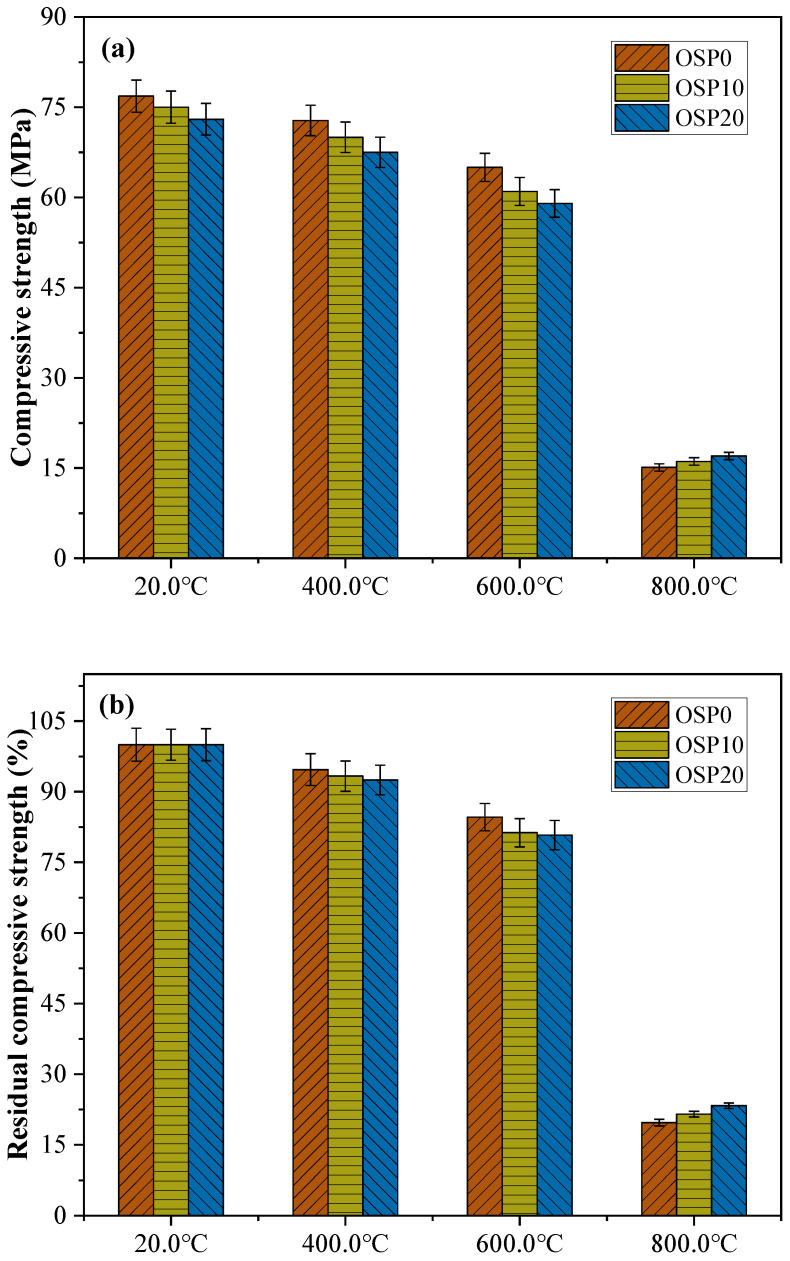
(**a**) The compressive strengths of OSP0, OSP10, and OSP20 samples cured for 180 d and exposed to 20.0, 400.0, 600.0, and 800.0 °C; (**b**) The percentage residual compressive strengths of OSP0, OSP10, and OSP20 samples exposed to 400.0, 600.0, and 800.0 °C.

By comparing the compressive strengths of the mixes before and after heating, the mechanical properties of the geopolymers were assessed. Figure 5a indicates the compressive strengths of OSP0, OSP10, and OSP20 samples cured for 180 d and exposed to 20.0, 400.0, 600.0, and 800.0 °C. Figure 5b shows the percentage residual compressive strengths of OSP0, OSP10, and OSP20 samples exposed to 400.0, 600.0, and 800.0 °C. The percentage of residual compressive strength can be calculated by Formula (1):

(1) $$\lambda_{x}=\frac{P_{y}}{P_{20}}\times 100\%$$

where $\lambda_{x}$ is the residual compressive strength, $P_{y}$ is the compressive strength at 20.0 °C, 400.0 °C, 600.0 °C, and 800.0 °C, and $P_{20}$ is the compressive strength at 20.0 °C.

The OSP content has different effects on the compressive strength of the mixture after passing through different temperatures. At 20.0 °C, the compressive strength of the mixture decreases with increasing OSP content. Although the TG curve indicated that more CASH gels were generated, this increase was insufficient to make up for the strength loss brought on by the decrease in GBFS and CP content. More concretely, the dilution effect resulted in a rise in the liquid-binder ratio and a loss in compressive strength when OSP took the place of GBFS and CP. In summary, the compressive strength was more severely decreased by the dilution impact than it was increased by adding additional CASH gels.

The strength of the mixture at 20.0 °C ranged from 73 to 77 MPa. The strength of all mixtures decreased with a gradual increase in temperature to 600.0 °C; however, the decrease was minimal. At 400 °C, the compressive strengths of OSP0, OSP10, and OSP20 are 72.8, 70, and 67.5 MPa, respectively. At 600.0 °C, the mixture OSP20 still exhibited a compressive strength above 59 MPa. This shows that below 600.0 °C, the effect of temperature increase on compressive strength is not obvious. Compressive strength experiences a fundamental change—a rapid drop—as the temperature rises, reaching 800.0 °C. Notably, unlike the mixtures exposed to 20.0, 400.0, and 600.0 °C, the mixtures exposed to 800.0 °C exhibited increased compressive strength with increasing OSP content. The compressive strengths of OSP0, OSP10, and OSP20 are 15.1, 16.1, and 17 MPa, respectively. This change in compressive strength is related to changes in several factors, including phase transitions and macroscopic shrinkage of reaction products (Section 3.4 and Section 3.6). Moreover, error bars were added to show the experimental data after processing. The coefficient of variance of the results for the strength and residual factor of compressive strength tests was less than 3.5%.

### 3.3. UPV

The stability of AAMs and cement concrete can be tested nondestructively using UPV testing [8,35]. A number of variables, including admixture, age, and liquid binder ratio, have an impact on UPV value [8,36,37]. This study considered the effects of two factors, OSP admixture and temperature, on the UPV of the mixture.

**Figure 6:**
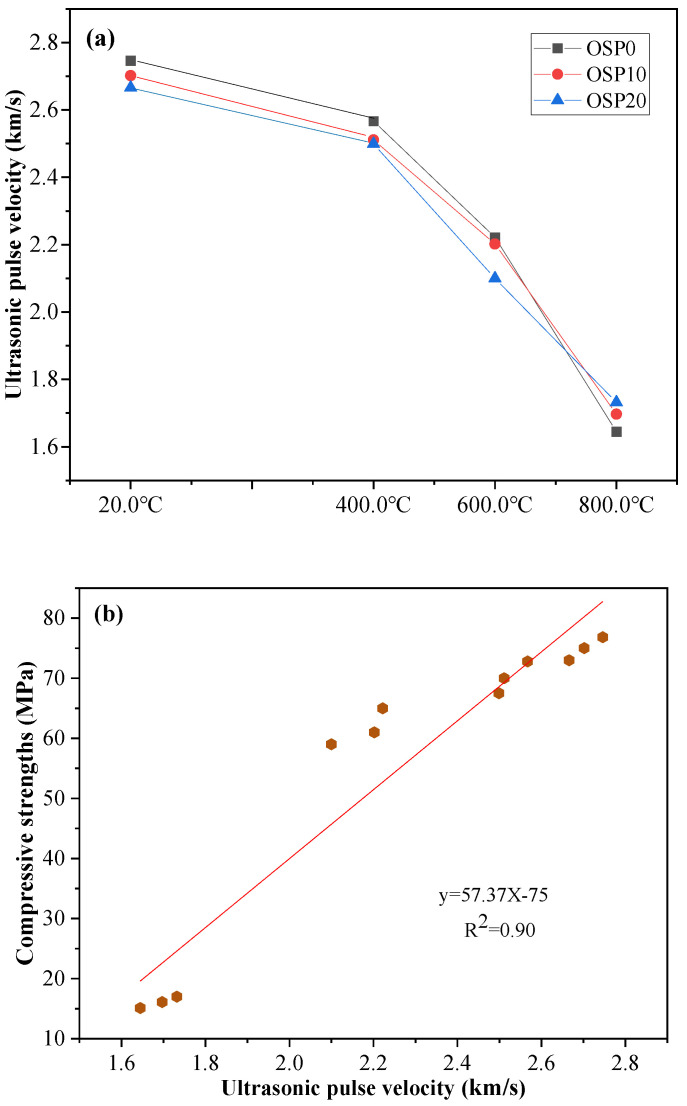
(**a**) UPV of OSP0, OSP10, and OSP20 samples at 180 days; (**b**) relationship between compressive strength and UPV.

The UPV test results of samples cured for 180 days and exposed to 20.0, 400.0, 600.0, and 800.0 °C are shown in Figure 6a. The UPV value of the mixture was affected by the amount of OSP added and temperature. In the control group without adding OSP, OSP0 had the highest UPV values of 20.0, 400.0, and 600.0 °C at 2.746, 2.567, and 2.222 km/s, respectively. At 20.0 °C, the values of the mixture range from 2.746 to 2.666 km/s. The value of UPV decreased with increasing OSP content. The main reason for the decrease in the UPV value is related to the dilution effect (decrease in the content of BFS and CP). Interestingly, when the temperature increased to 800.0 °C, the value of UPV tended to increase with an increasing amount of OSP added. The UPV values of the OSP0, OSP10, and OSP20 samples were 1.645 km/s, 1.697 km/s, and 1.732 km/s, respectively. The reason for the rising trend of UPV is related to the different phase transitions of the reaction products of OSP0, OSP10, and OSP20 samples exposed to 800.0 °C (See Section 3.6 for the different species of substances produced by phase transitions).

In addition, by carefully observing the test results of the compressive strength and UPV, it was found that the development trends of the UPV and strength of the OSP0, OSP10, and OSP20 samples at different temperatures were similar. The UPV and compressive strengths were compared, as shown in Figure 6b. At different temperatures, with the increase in OSP content, the strength and UPV of the OSP0, OSP10, and OSP20 samples exhibited a similar development trend. To further analyze the correlation between the UPV and compressive strength of the OSP0, OSP10, and OSP20 samples, a correlation analysis between the compressive strength and UPV of the OSP0, OSP10, and OSP20 samples exposed to different temperatures was carried out, as shown in Figure 6b. The strengths of the OSP0, OSP10, and OSP20 samples exhibited a good linear relationship with UPV, and the correlation factor, R^2^, was 0.90.

### 3.4. XRD

**Figure 7:**
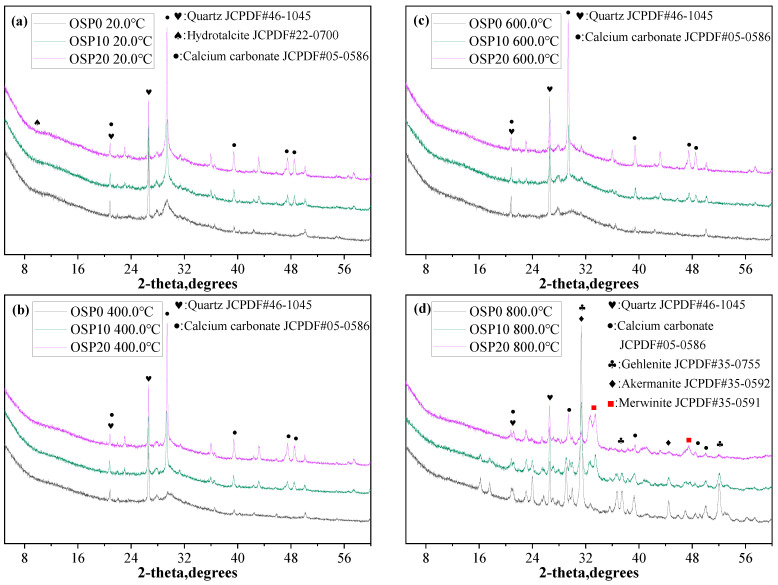
OSP0, OSP10, and OSP20 samples’ 180-day XRD patterns; (**a**): 20.0 °C, (**b**): 400.0 °C, (**c**): 600.0 °C, and (**d**): 800.0 °C.

The shape of semi-crystalline and crystalline peaks of AAM reaction products can be examined using XRD. Figure 7 shows the XRD spectra of OSP0, OSP10, and OSP20 samples exposed to different temperatures. Among the alkali-activated products, quartz, calcium carbonate, hydrotalcite phase crystals, and semi-crystalline CASH phases were detected. Between 26° and 27° is the peak of quartz. This was because of the addition of ceramic powder, and quartz was the main crystal phase of the ceramic powder. In alkali-activated binary and ternary mixtures, quartz is always present because of its stability and reaction inertia. Between 29° and 30°, the hump of the CASH and the spike of calcium carbonate overlap. The spike in calcium carbonate increased with increasing OSP content. Hydrotalcite phases are confirmed based on the results shown in reference [28]. In addition, the main products of geopolymers are amorphous CASH and NASH, in which the content of crystalline hydrotalcite is very limited. It is necessary to combine various experimental methods to judge the existence of small content of crystalline hydrotalcite.

During the heating test, the hydration products of the OSP0, OSP10, and OSP20 samples underwent decomposition and phase transition. The hump of the CASH gradually smoothed out as the temperature rose from 20 °C to 800.0 °C, and it eventually vanished at 800.0 °C. This indicates that the semi-crystalline CASH phase, dehydration, and decomposition occurred [38]. In the OSP10 and OSP20 samples containing OSP, the peak intensity of calcium carbonate decreased as the temperature rose from 400.0 °C to 800.0 °C. However, the peak of calcium carbonate can still be detected at 800.0 °C. This indicated that CaCO_3_ in the OSP10 and OSP20 samples was not completely decomposed when the temperature reached 800.0 °C.

When OSP0, OSP10, and OSP20 samples were heated to 800.0 °C, the number and shape of the mixture peaks changed significantly, and new sharp peaks appeared. This indicates that some amorphous and semi-crystalline phases in the reaction products changed into crystalline phases, accompanied by the formation of new substances. The presence of new crystalline phases, gehlenite [Ca_2_Al_2_SiO_7_] and akermanite [Ca_2_MgSi_2_O_7_], was detected in the OSP0sample after exposure to 800.0 °C. This is in line with the findings that have been published in the literature [39,40]; the phase transformation of alkali-activated slags exposed to high temperatures produces gehlenite and akermanite. It is worth noting that the presence of crystalline phases gehlenite and akermanite was not detected in the OSP20 sample added with 20% OSP, but a new merwinite [Ca_3_Mg(SiO_4_)_2_] crystalline phase appeared. This suggests that the addition of OSP inhibits gehlenite and akermanite growth and promotes merwinite formation. A careful study found that the calcium content of merwinite was higher than that of gehlenite and akermanite. This is because the addition of OSP provides more calcium for the phase transition to the merwinite crystalline phase. The generation of different crystalline phases is the main reason for subtle changes in the macroscopic properties.

According to the experimental results of compressive strength, the phase transition to producing new substances is not conducive to the development of mechanical properties [39]. However, when the samples were exposed to 800 °C, the compressive strength increased with the increase of OSP content. This shows that the composition of the new material produced by the phase transition has different contributions to the mechanical properties. The contribution of the crystalline phases gehlenite, akermanite and merwinite to the mechanical properties requires further investigation.

### 3.5. FTIR

**Figure 8:**
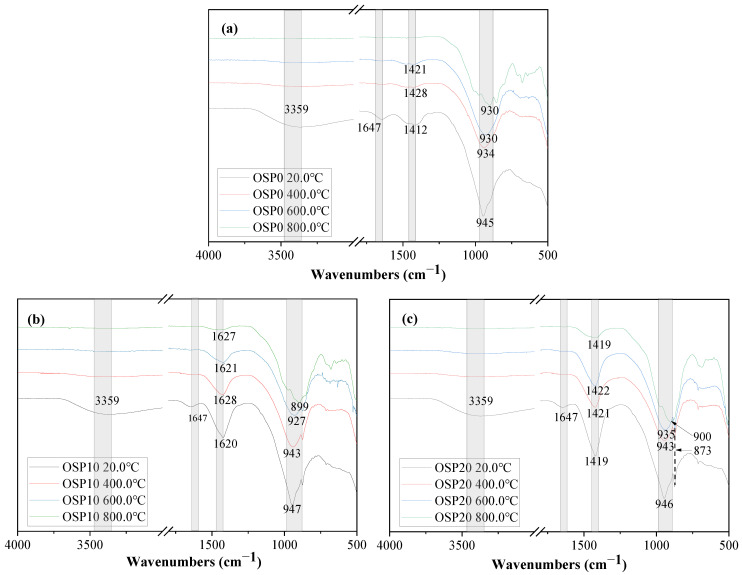
FTIR spectra of samples at 20.0 °C, 400.0 °C, 600.0 °C, and 800.0 °C at 180 days; (**a**): OSP0, (**b**): OSP10, and (**c**): OSP20.

The FTIR spectra of OSP0, OSP10, and OSP20 samples heated to different temperatures are reported in Figure 8. At 20.0 °C, the absorption peak caused by the stretching vibration of the O-H bond appears near wave number 3359 cm^−1^, while the absorption peak caused by the bending vibration of the O-H bond appears near wave number 1647 cm^−1^ [41,42]. At about 1421 cm^−1^, the absorption peak of the O-C bond can be observed. With increasing OSP content, the O-C bond absorption peak’s intensity rises. The OSP’s calcium carbonate content caused this. Absorption peaks caused by asymmetric stretching vibrations of O–Si(Al) bonds in CASH gels appear in the wavenumber region of 998–937 cm^−1^ [43,44,45].

The stretching and bending vibration peaks associated with O-H bonds vanished when the exposure temperature of the mixture was raised from 20.0 °C to 400.0 °C, suggesting substantial dehydroxylation in each phase, including CASH. The weakening of the intensity of the N–O-M (M =Si; N=Al) bond absorption peak at 945 cm^−1^ is particularly pronounced and shifts toward the lower wave number. This situation indicates that the aluminosilicate network structure in the CASH gels becomes loose.

As the exposure temperature of the mixture increased from 400.0 °C to 600.0 °C, the intensity of the O-C bond absorption peak gradually decreased, suggesting ongoing calcium carbonate decomposition. However, no significant changes were observed in the intensity of the N-O-M bond absorption peak. This shows that the temperature change at this stage had little effect on the change in the aluminosilicate network structure.

Absorption peaks due to O-C bonding were detected in the OSP10 and OSP20 samples containing OSP when the mixture was exposed to 800.0 °C. This indicated that calcium carbonate was not completely decomposed after exposure to 800.0 °C. The shape, intensity, and wavenumber of the N–O–M bond absorption peaks changed significantly. This implies a significant change in the network structure of aluminum in CASH gels. Combined with the XRD analysis, a phase transition occurred at this temperature. This is the reason for the sharp decrease in the mechanical properties of the mixture [39].

### 3.6. Size Changes and Image

**Figure 9:**
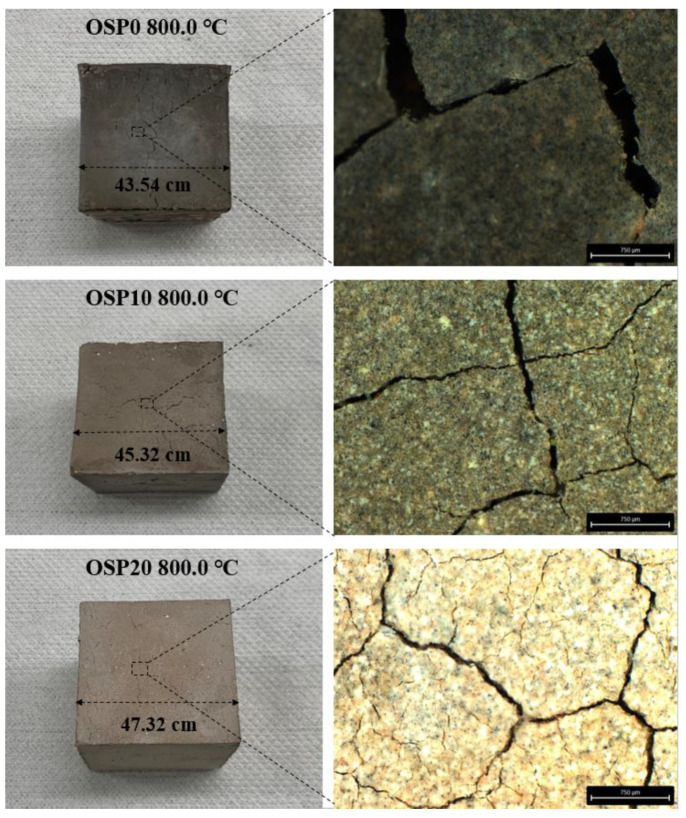
Macro image of OSP0, OSP10, and OSP20 samples at 180 days.

Figure 9 shows the macroscopic and mesoscopic images of OSP0, OSP10, and OSP20 samples exposed to 800.0 °C for one hour. Careful analyses reveal that the colors of the mixture’s macroscopic and mesoscopic images are not exactly the same. This is because of the differences in the focusing and imaging of the microscope. When exposed to 800.0 °C, the macroscopic color of the mixture changed, and cracks appeared. OSP0 is dark green because the color of CP is overwritten by the new material color produced by the phase transition; OSP20 is pale yellow. This is the result of the joint action of the new substances produced by the phase transition, the CaO produced by the decomposition of OSP, and the raw material CP.

**Figure 10:**
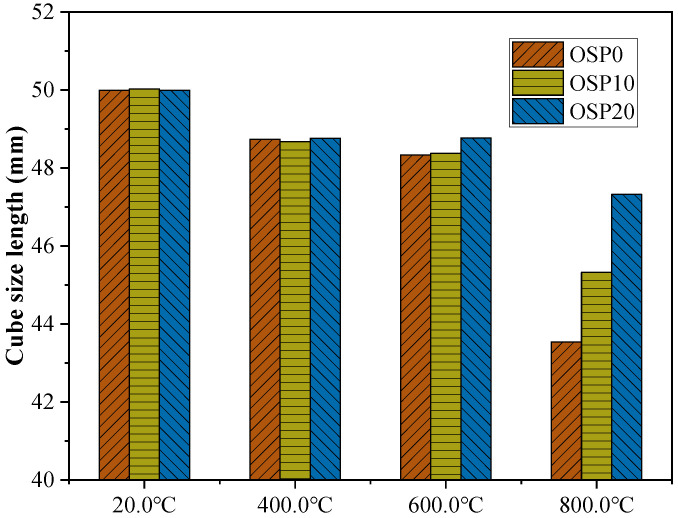
Size of OSP0, OSP10, and OSP20 samples at 180 days.

Figure 10 shows the dimensional changes in the OSP0, OSP10, and OSP20 samples exposed to different temperatures. All mixtures shrink when the temperature rises from 20.0–600.0 °C. This is caused by CASH dehydration and partial decomposition. The difference in shrinkage-induced dimensional changes was insignificant for the mixtures exposed to 400.0 °C and 600.0 °C. When the temperature rose from 600.0 °C to 800.0 °C, the mixture shrank sharply with the increase in the OSP content, and the difference in the size change caused by shrinkage was more obvious. The side lengths of OSP0, OSP10, and OSP20 were 43.54, 45.32, and 47.32 cm, respectively. This was caused by the microconstraint effect [46]. The microscopic confinement effect is that when the temperature rises from 600.0 °C to 800.0 °C, the phase transition occurs after the decomposition of CASH, resulting in a massive change in the macroscopic size. After the addition of OSP, OSP was relatively stable at high temperatures, but part of the OSP decomposed and generated CaO after decomposition. Undecomposed OSP and decomposed CaO constrained the deformation caused by the decomposition phase transition of CASH. Compared with OSP20 containing OSP, the surface of OSP0 became more uneven. This also shows that the addition of OSP suppresses the uneven shrinkage of the mixture.

## 4. Discussion 

The third section investigates the macro- and micro-property results of AAM with OSP added. This section discusses the differences and similarities between the results of previous studies and ours.

Most of the previous studies on the use of oyster shell powder (OSP) as a building material focused on the effect on the properties of cement concrete when used as a supplementary cementitious material instead of cement [16,17]. Han et al. [16] studied the feasibility of OSP and GBFS as cement substitutes to prepare sustainable concrete. They found that adding OSP accelerated the hydration reaction in the first 24 h of the mixture. Her et al. [17] carried out research on OSP cement as a substitute for limestone. They found that OSP was a suitable substitute for limestone.

Our research focuses on the effect of adding OSP on the high-temperature resistance of alkali-stimulated GBFS and CP-based polymers. When the samples were exposed to 800 °C, the compressive strength increased with increasing OSP content. This indicates that after the addition of OSP, the composition of the new crystalline phase produced by the phase transformation contributes differently to the mechanical properties.

## 5. Conclusions

This study investigated the high-temperature performance of a three-basic AAM made of recycled GBFS, CP, and OSP after curing for 180 d. The scientific innovation of this paper mainly includes the following. First, the research results of this paper provide a solution for the application of environment-friendly building materials. Second, most past studies have focused on the effect of OSP on the properties of cement concrete. The research of this paper focuses on the impact of OSP on the performance of AAM. Third, past studies have focused on room-temperature performance, while this paper investigates the high-temperature resistance of alkali-activated GBFS and CP with the addition of OSP.

The scientific results of this paper mainly include the following points:

1. The TG analysis indicated that the addition of OSP to the mixture, as compared to the control OSP0, increased CASH gel production, along with the observation of a decomposition peak of calcium carbonate. This could be attributed to the participation of some calcium carbonates in OSP in the alkali activation reaction, leading to the formation of CASH gel. Furthermore, the remaining calcium carbonate decomposed at high temperatures.
2. As the temperature increased from 20 °C to 600.0 °C, a decline in the compressive strength of the mixture was observed. This is attributed to the dehydration and partial decomposition of the CASH gel. Subsequently, a significant decrease in compressive strength was observed as the temperature increased from 600.0 °C to 800.0 °C. This can be attributed to the mixture’s phase transition and uneven shrinkage.
3. At 20.0 °C, the addition of OSP resulted in lower UPV values for OSP10 and OSP20 compared to the control group OSP0, which did not have OSP added. This observation may be attributed to the dilution effects caused by OSP. Furthermore, a robust correlation was observed between UPV and compressive strength, with a correlation factor (R^2^) of 0.90.
4. The XRD pattern shows that new crystalline peaks (gehlenite and akermanite) appear in the mixture when the temperature increases from 600.0 °C to 800.0 °C. In addition, the morphology and position of the peaks of OSP20 were changed compared to the control OSP0. This is because, at 800.0 °C, the mixture undergoes a phase transition, and the substance produced by the phase transition also changes.
5. Based on the FTIR analysis, the intensity of the N-O-M bond absorption peak experiences a notable reduction as the temperature rose from 20.0 °C to 400.0 °C. This suggests that the aluminosilicate network structure of CASH gel becomes less dense.
6. The mixture’s color altered, and the shrinkage grew as the temperature rose from 600.0 °C to 800.0 °C. This is because the mixture underwent a phase transition, and the OSP20 sample containing OSP decomposed to produce off-white CaO.

In addition, in further research, more work should be conducted to clarify the influences of other substances containing calcium carbonate (such as calcium carbonate minerals) on the high-temperature resistance of alkali-activated materials.

## Figures and Tables

**Table 1:** Chemical compositions of raw materials (GBFS, CP, and OSP) in this study.

Raw Materials (wt.%)	SiO_2_	Al_2_O_3_	CaO	Fe_2_O_3_	MgO	Na_2_O	K_2_O	ZnO	SO_3_	LOI ^x^
GBFS	32.2	15.7	38.9	0.65	7.08	0.30	0.61	-	0.65	1.25
CP	66.1	15.9	9.32	2.38	0.58	1.12	1.93	0.15	0.42	1.19
OSP	0.70	-	54.82	0.15	0.51	0.58	0.17	-	-	43.07

^x^ Loss on ignition.

**Table 2:** Paste samples mixtures.

Mix NO.	GBFS	CP	OSP	NaOH	Water	Water-Glass	L/(BFS + CP)	L/B
OSP0	80	20	0	3.2	30.8	16	0.5	0.5
OSP10	72	18	10	3.2	30.8	16	0.556	0.5
OSP20	64	16	20	3.2	30.8	16	0.625	0.5

## Data Availability

The data presented in this study are available from the corresponding author upon reasonable request.

## References

[1] Cloete S., Giuffrida A., Romano M.C., Zaabout A. (2020). Economic assessment of the swing adsorption reactor cluster for CO_2_ capture from cement production. J. Clean. Prod..

[2] Zhang G.-Y., Lin R.-S., Wang Y.-S., Wang X.-Y. (2022). Influence of K^+^ and CO_3_^2−^ in activator on high-temperature performance of alkali-activated slag-ceramic powder binary blends. Case Stud. Constr. Mater..

[3] Han Y., Lin R. (2022). Sustainable Mixtures Using Waste Oyster Shell Powder and Slag Instead of Cement: Performance and Multi-Objective Optimization Design. Constr. Build. Mater..

[4] Pławecka K., Bazan P., Lin W.-T., Korniejenko K., Sitarz M., Nykiel M. (2022). Development of Geopolymers Based on Fly Ashes from Different Combustion Processes. Polymers.

[5] Hwang C.-L., Yehualaw M.D., Vo D.-H., Huynh T.-P. (2019). Development of high-strength alkali-activated pastes containing high volumes of waste brick and ceramic powders. Constr. Build. Mater..

[6] Damtoft J.S., Lukasik J., Herfort D., Sorrentino D., Gartner E.M. (2008). Sustainable development and climate change initiatives. Cem. Concr. Res..

[7] El-Gamal S., El-Hosiny F., Amin M., Sayed D. (2017). Ceramic waste as an efficient material for enhancing the fire resistance and mechanical properties of hardened Portland cement pastes. Constr. Build. Mater..

[8] Zhang G.-Y., Bae S.-C., Lin R.-S., Wang X.-Y. (2021). Effect of Waste Ceramic Powder on the Properties of Alkali–Activated Slag and Fly Ash Pastes Exposed to High Temperature. Polymers.

[9] Zhang G.-Y., Ahn Y.-H., Lin R.-S., Wang X.-Y. (2021). Effect of Waste Ceramic Powder on Properties of Alkali-Activated Blast Furnace Slag Paste and Mortar. Polymers.

[10] Sun Z., Cui H., An H., Tao D., Xu Y., Zhai J., Li Q. (2013). Synthesis and thermal behavior of geopolymer-type material from waste ceramic. Constr. Build. Mater..

[11] Ruviaro C.F., Gianezini M., Brandão F.S., Winck C.A., Dewes H. (2012). Life cycle assessment in Brazilian agriculture facing worldwide trends. J. Clean. Prod..

[12] Barros M., Bello P., Bao M., Torrado J. (2009). From waste to commodity: Transforming shells into high purity calcium carbonate. J. Clean. Prod..

[13] Seo J.H., Park S.M., Yang B.J., Jang J.G. (2019). Calcined oyster shell powder as an expansive additive in cement mortar. Materials.

[14] H Silva T., Mesquita-Guimarães J., Henriques B., Silva F.S., Fredel M.C. (2019). The potential use of oyster shell waste in new value-added by-product. Resources.

[15] Yang E.-I., Yi S.-T., Leem Y.-M. (2005). Effect of oyster shell substituted for fine aggregate on concrete characteristics: Part I. Fundamental properties. Cem. Concr. Res..

[16] Han Y., Lin R., Wang X.-Y. (2021). Performance of sustainable concrete made from waste oyster shell powder and blast furnace slag. J. Build. Eng..

[17] Her S., Park T., Zalnezhad E., Bae S. (2021). Synthesis and characterization of cement clinker using recycled pulverized oyster and scallop shell as limestone substitutes. J. Clean. Prod..

[18] Mo K.H., Alengaram U.J., Jumaat M.Z., Lee S.C., Goh W.I., Yuen C.W. (2018). Recycling of seashell waste in concrete: A review. Constr. Build. Mater..

[19] Liao Y., Fan J., Li R., Da B., Chen D., Zhang Y. (2022). Influence of the usage of waste oyster shell powder on mechanical properties and durability of mortar. Adv. Powder Technol..

[20] Song Q., Wang Q., Xu S., Mao J., Li X., Zhao Y. (2022). Properties of water-repellent concrete mortar containing superhydrophobic oyster shell powder. Constr. Build. Mater..

[21] Liao Y., Wang X., Wang L., Yin Z., Da B., Chen D. (2022). Effect of waste oyster shell powder content on properties of cement-metakaolin mortar. Case Stud. Constr. Mater..

[22] Firdous R., Hirsch T., Klimm D., Lothenbach B., Stephan D. (2021). Reaction of calcium carbonate minerals in sodium silicate solution and its role in alkali-activated systems. Miner. Eng..

[23] Gao X., Yu Q., Brouwers H. (2015). Properties of alkali activated slag–fly ash blends with limestone addition. Cem. Concr. Compos..

[24] Nasaeng P., Wongsa A., Cheerarot R., Sata V., Chindaprasirt P. (2022). Strength enhancement of pumice-based geopolymer paste by incorporating recycled concrete and calcined oyster shell powders. Case Stud. Constr. Mater..

[25] Yang B., Jang J.G. (2020). Environmentally benign production of one-part alkali-activated slag with calcined oyster shell as an activator. Constr. Build. Mater..

[26] Djobo Y., Elimbi A., Manga J.D., Ndjock I.D.L. (2016). Partial replacement of volcanic ash by bauxite and calcined oyster shell in the synthesis of volcanic ash-based geopolymers. Constr. Build. Mater..

[27] Huseien G.F., Sam A.R.M., Mirza J., Tahir M.M., Asaad M.A., Ismail M., Shah K.W. (2018). Waste ceramic powder incorporated alkali activated mortars exposed to elevated Temperatures: Performance evaluation. Constr. Build. Mater..

[28] Huseien G.F., Sam A.R.M., Shah K.W., Mirza J. (2020). Effects of ceramic tile powder waste on properties of self-compacted alkali-activated concrete. Constr. Build. Mater..

[29] Han Y., Lin R., Wang X.-Y. (2023). Preparation of Nano Calcite by the Carbon Capture Technology to Improve the Performance of Ultrahigh-Performance Concrete Containing Calcined Clay. ACS Sustain. Chem. Eng..

[30] Han Y., Lin R., Wang X.-Y. (2022). Carbon conversion technology for performance improvement and environmental benefits of ultra-high-performance concrete containing slag. J. Mater. Res. Technol..

[31] Gao X., Yu Q., Brouwers H. (2015). Reaction kinetics, gel character and strength of ambient temperature cured alkali activated slag–fly ash blends. Constr. Build. Mater..

[32] Kim M.S., Jun Y., Lee C., Oh J.E. (2013). Use of CaO as an activator for producing a price-competitive non-cement structural binder using ground granulated blast furnace slag. Cem. Concr. Res..

[33] Lin R.-S., Han Y., Wang X.-Y. (2021). Macro-meso-micro experimental studies of calcined clay limestone cement (LC3) paste subjected to elevated temperature. Cem. Concr. Compos..

[34] Coppola B., Palmero P., Montanaro L., Tulliani J.-M. (2020). Alkali-activation of marble sludge: Influence of curing conditions and waste glass addition. J. Eur. Ceram. Soc..

[35] Acharya P.K., Patro S.K. (2015). Effect of lime and ferrochrome ash (FA) as partial replacement of cement on strength, ultrasonic pulse velocity and permeability of concrete. Constr. Build. Mater..

[36] Wang C.-C., Wang H.-Y. (2017). Assessment of the compressive strength of recycled waste LCD glass concrete using the ultrasonic pulse velocity. Constr. Build. Mater..

[37] Jiang H., Yi H., Yilmaz E., Liu S., Qiu J. (2020). Ultrasonic evaluation of strength properties of cemented paste backfill: Effects of mineral admixture and curing temperature. Ultrasonics.

[38] Park S.M., Jang J.G., Lee N., Lee H.-K. (2016). Physicochemical properties of binder gel in alkali-activated fly ash/slag exposed to high temperatures. Cem. Concr. Res..

[39] Cai R., Ye H. (2021). Clinkerless ultra-high strength concrete based on alkali-activated slag at high temperatures. Cem. Concr. Res..

[40] Rovnaník P., Bayer P., Rovnaníková P. (2013). Characterization of alkali activated slag paste after exposure to high temperatures. Constr. Build. Mater..

[41] Ylmén R., Jäglid U., Steenari B.-M., Panas I. (2009). Early hydration and setting of Portland cement monitored by IR, SEM and Vicat techniques. Cem. Concr. Res..

[42] Han Y., Lin R., Wang X.-Y. (2021). Performance and sustainability of quaternary composite paste comprising limestone, calcined Hwangtoh clay, and granulated blast furnace slag. J. Build. Eng..

[43] Nguyen H.-A., Chang T.-P., Shih J.-Y., Chen C.-T. (2019). Influence of low calcium fly ash on compressive strength and hydration product of low energy super sulfated cement paste. Cem. Concr. Compos..

[44] Barnett S., Macphee D.E., Lachowski E.E., Crammond N. (2002). XRD, EDX and IR analysis of solid solutions between thaumasite and ettringite. Cem. Concr. Res..

[45] Lin R.-S., Lee H.-S., Han Y., Wang X.-Y. (2021). Experimental studies on hydration–strength–durability of limestone-cement-calcined Hwangtoh clay ternary composite. Constr. Build. Mater..

[46] Guo G., Lv C., Liu J., Wang L. (2022). Properties of Fiber-Reinforced One-Part Geopolymers: A Review. Polymers.

